# Empowering leadership and frontline employees’ emotional labor: the mediation effects of job passion

**DOI:** 10.3389/fpsyg.2025.1437736

**Published:** 2025-04-08

**Authors:** Pengfei Cheng, Linfei Zhou, Tong Liu, Na Ge

**Affiliations:** School of Economics and Management, Xi’an University of Technology, Xi’an, China

**Keywords:** empowering leadership, job passion, frontline employee, deep acting, surface acting

## Abstract

**Instruction:**

In order to deliver superior service experience to customers, frontline employees must regulate their emotional expressions during service encounters. This study examines how empowering leadership influences emotional labor (deep acting and surface acting) through the mediating role of job passion.

**Methods:**

Using data of 1,040 frontline employees across three service industries, the proposed mediating model was tested.

**Results:**

The findings revealed that: empowering leadership predicted deep acting and reduced surface acting. Job passion mediated the relationship between empowering leadership and emotional labor. Specifically, empowering leadership influenced surface acting only through obsessive passion. Empowering leadership had a “double-edged” effect on deep acting, operating through both harmonious and obsessive passion simultaneously.

**Discussion:**

This study highlights the mediating role of job passion in translating empowering leadership into emotional labor strategies. The findings help service organizations refine leadership strategies to enhance emotional regulation in frontline service roles.

## 1 Introduction

The rise of the competition has underscored the pivotal role of the service experience as a determinant of the competitiveness of service organizations (Zou et al., 2020). To create an excellent customer service experience, frontline employees are expected to regulate and express appropriate emotions during service encounters. In her exploratory research, Hochschild (1983) defined flight attendants’ act of managing their emotional expression in terms of emotional labor. There are two different strategies for service workers to regulate their emotions: deep acting (altering their inner feelings to display authentic emotions) and surface acting (displaying fake emotions) (Ashforth and Humphrey, 1993; Grandey, 2003). Previous studies have shown that deep acting was prone to lead to desirable outcomes, including employee performance (Alsakarneh et al., 2019), service quality (Wang, 2020), and customer satisfaction (Gong et al., 2020). While surface acting tends to result in undesirable outcomes and impair the wellbeing of employees (Riforgiate et al., 2022). Therefore, it is widely accepted in both academia and practice that service organizations should guide service workers to take advantage of deep acting, as opposed to surface acting, during service interactions.

Given the significance of emotional labor in organizational contexts, a considerable amount of literature has been devoted to exploring various antecedents of emotional labor. Leaders, who represent the service organization to interact directly with frontline employees, were expected to influence frontline employees’ job behaviors (Dong et al., 2022). Prior search has supported that transformational leadership (Cheng et al., 2023), ethical leadership (Mo and Shi, 2017), and servant leadership (Kahya and Kuloğlu, 2022; Lu et al., 2019) resulted in different emotional labor strategies. During dynamic service encounters, it is particularly important that frontline employees who interact directly with customers are afforded sufficient autonomy to respond promptly and effectively to customer needs. Therefore, growing leaders of service organization delegate power to their employees (Lim, 2021), which is defined as empowering leadership. Especially in China, with the fast growth of the service sector and the introduction of Western management theory, empowering leadership has become increasingly popular in Chinese service organizations. Although the influences of empowering leadership on desirable employee outcomes including voice (Wang et al., 2020), proactive behavior (Dong et al., 2022), knowledge sharing (Burhan and Khan, 2024) and creativity (Peng et al., 2023) has been confirmed by previous studies, surprisingly, to date, few studies have examined whether leaders’ empowering behaviors might have an effect on how subordinates regulate their emotions during service interactions. Therefore, positing and empirically examining the influence of empowering leadership on emotional labor is the first objective of this study.

Given the discretionary nature of emotional labor (Cossette, 2014), whether frontline employee regulates emotions through deep or surface acting is determined by his/her intronic motivation. Therefore, it is necessary to unveil the psychological mechanism through which empowering leadership is internalized by frontline employees and in turn affects their emotional labor. Furthermore, in their review of empowering leadership, Kim et al. (2018) also called for uncovering possible psychological processes that could interpret how empowering leadership predicts followers’ job behaviors. The second objective is to uncover how leaders’ empowering behavior affects subordinates’ emotional labor by introducing mediators.

Previous researches have predominantly employed the Social Exchange Theory (Chow, 2018; Rai and Kim, 2021) or the Leader-member exchange (LMX) theory (Horoub and Zargar, 2022; Lee et al., 2018) to explain how does empowering leadership affects employees’ job behaviors (e.g., OCB, creativity and voice). As the foundational element of Social Exchange Theory and Leader-member exchange (LMX) theory, the norm of reciprocity makes individuals felt obligation to return favors, gifts, or services they have received during their social interactions (Burger et al., 2009). In service industry, when frontline employees are empowered, they are expected to reciprocate their leaders and organizations by engaging in desirable behaviors that toward their leaders, coworkers and organizations. However, as a boundary-spanner, the typical activities of frontline employees in their workplace target customers, such as emotional labor. Therefore, the norm of reciprocity is not a suitable interpretative framework for understanding why subordinates choose distinct strategies to regulate their emotions. In response to these theoretical gaps, this study seeks to unveil the process of empowering leadership to emotional labor from a motivational perspective. As a motivational construct, Vallerand et al. (2003) defined job passion as one’s profound inclination to her/his job. Passionate individuals might derive enjoyment from their work and voluntarily invest efforts in their role. Consequently, previous research has relied on job passion to uncover the process of how employees internalize workplace factors, such as leadership, and thereby affect job role behaviors (Zigarmi et al., 2009). Hence, the current research thirdly aims to interpret the mechanism of empowering leadership on subordinates’ emotion-regulating strategies in terms of a motivational aspect, rather than a normative reciprocity aspect.

In order to fill the aforementioned theoretical gaps, we plan to investigate the mechanism through which empowering leadership affects emotional labor by focusing on job passion’s mediating effects. Three contributions are made. First, we could make contributions to leadership theory through identifying unexplored outcomes associated with empowering leadership. In response to Kim et al. (2018) call for exploring the influence of empowering leadership on subordinates’ emotions, the present research examined the direct link of empowering leadership to emotional labor. By doing so, we were able to supply support for the view that the effects of leadership could not only reach subordinates but also spill over to customers (Ahearne et al., 2005). Second, the introduction of job passion as a mediator contributes to emotional labor theory by enhancing our knowledge of the motivational process by that empowering leadership influences frontline employees’ strategies of regulating their emotions. Although the importance of emotional labor has been recognized by academics and practitioners, it remains difficult for service managers to directly intervene in frontline employees’ emotion regulation strategies during service encounters. Our findings may supply service practitioners with management tools to indirectly influence frontline employees’ emotional labor by evoking their job passion. Finally, unlike previous literature that relied on the norm of reciprocity to interpret why leaders could affect subordinates’ behavior, we proposed from a motivational perspective that job passion would mediate the link of empowering leadership to emotional labor. This work would broaden the theoretical foundation for interpreting how empowering leadership influences frontline employees’ behaviors toward external stakeholders (customers).

## 2 Theoretical development and hypothesis

### 2.1 Empowering leadership and emotional labor

Scholars describe empowering leadership as a series of leadership behaviors that allocate power and autonomy to subordinates (Pangarso et al., 2022; Sharma and Kirkman, 2015). Typical empowering behaviors include coaching, sharing authority, encouraging followers self-development and taking responsibility (Amundsen and Martinsen, 2014). Essentially, empowering leader behaviors enhance employee motivation by delcegating authority and responsibility to subordinates, allowing them to perform their job in a self-directed manner (Conger and Kanungo, 1988). To differentiate empowering leadership from other similar leadership styles, Cheong et al. (2019) compared empowering leadership with participative leadership, transformational leadership, ethical leadership and leader-member exchange (LMX). They argued that empowering leadership is a unique and independent leadership style. An existing body of research evidence showed that empowering leadership could predict subordinates’ proactive behaviors such as OCB (Wang et al., 2023), voice (Raub and Robert, 2012), creativity (Liu et al., 2024) and work engagement (Arshad et al., 2022), which means that empowering leadership mostly results in more desirable consequences.

Given the dynamic and interactive nature of service encounters (Ma and Dubé, 2011), frontline employees must have sufficient autonomy to deal effectively with customer requests. Therefore, empowering leadership, characterized by autonomy, is increasingly common in service organizations (Aryee et al., 2019). As the most cited theoretical basis of emotional labor strategies, Conservation of Resources (COR) theory posits that individuals actively strive to obtain, protect and construct precious resources and minimize any threats of resource loss (Hobfoll et al., 2018). Following this theory, employees with adequate resources tend to conduct behaviors that deplete more resources, and vice versa. Under the empowerment condition, employees with high job autonomy and authority are expected to have high levels of job resources (Trépanier et al., 2014). Similarly, Kim and Beehr (2023) also argued that empowering leadership could effectively improve subordinates’ job resources. Since frontline employees must spend lots of psychological resources in performing emotional labor during service interactions, it is reasonable to infer that the form of emotional labor used by frontline employees depends on their level of job resources. Cheng et al. (2022) have pointed out that engaging in deep acting depleted much more resources than surface acting. According to their view, empowered employees with high autonomy are expected to regulate their emotions by devoting more efforts to adjusting their inner felt emotions (deep acting) instead of merely expressing fake emotions (surface acting).

Furthermore, prior studies have indicated that empowering leadership foster subordinates’ psychological empowerment (Hoang et al., 2021). High psychological empowerment can make employees more enthusiastic (Meng and Sun, 2019) and enhance the meaning of work (Livne and Rashkovits, 2018). Under these conditions, positive emotions that meet the expectations of the service company tend to be aroused in frontline employees. Li et al. (2021) have reported that high psychological empowerment can support frontline employees actively change their emotions and behaviors by using deep rather than surface acting. Taken together, we propose:

Hypothesis 1: Empowering leadership exerts a positive influence on deep acting.

Hypothesis 2: Empowering leadership exerts a negative influence on surface acting.

### 2.2 The mediation effects of job passion

Employees with high job passion consider her/his job to be valuable, enjoyable, and worthy of devoting efforts to perform (Vallerand et al., 2003). Based on Self-determination theory (SDT), which posits that the extent of people’s needs for autonomy, competence, and relatedness are met determines people’s behavior (Ryan and Deci, 2000), Vallerand et al. (2003) argued that the degree of employees’ basic psychological needs are met predicts employees’ different types of job motivations (autonomous vs. controlled), which further evoke distinct types of job passion (harmonious vs. obsessive). Specifically, harmonious passion emerges when employees autonomously internalize the role of their job into their identity. Harmonious passionate employees voluntarily invest efforts or resources in their job for enjoyment or challenge rather than out of a sense of obligation. Consequently, many previous studies have provided evidence that harmonious passion would predict employees’ positive behaviors and wellbeing, such as job satisfaction (Peixoto et al., 2023), innovation behavior (Luu, 2019), job engagement (Teng et al., 2021) and greater wellbeing (Tolentino et al., 2022).

In contrary, when employees internalize the role of their job in a controlled way, obsessive passion emerges. Obsessive passionate employees engage in their job under some specific conditions, such as for higher salaries, promotion and enhancing self-esteem. Under these conditions, relevant research has indicated that obsessive passion would lead to undesirable consequences for both employees and organization, such as burnout (Landay et al., 2022), low performance (Chummar et al., 2019) and unethical behavior (Hillebrandt and Barclay, 2022).

#### 2.2.1 Empowering leadership and job passion

Following Self-Determination Theory, whether harmonious or obsessive passion is aroused depends on the degree of people’s needs for autonomy, competence, and relatedness are met (Ryan and Deci, 2000). For employees whose psychological needs are well met, harmonious passion is evoked and they can derive enjoyment from their work. Otherwise, employees with unmet psychological needs are “forced” to complete the work with obsessive passion.

For frontline employees in service organizations, their daily tasks consist of providing services and interacting with customers under the supervision of their leaders. Therefore, the leadership style they face determines the satisfaction of employees’ psychological needs (Deci et al., 2017). First, empowering leadership provides subordinates with more autonomy, which is not only necessary to ensure high service quality, but also to satisfy psychological needs (Geralis and Terziovski, 2003). Similarly, Fernet et al. (2014) have found people with high autonomy tend to be harmonious passionate. Second, empowering leader behavior involves distributing power to followers and trusting them to achieve superior performance, which implies leader’ acknowledgement of the competence of employees. Cheong et al. (2016) further argued that when subordinates were empowered, they tended to have high self-efficacy. Under these conditions, frontline employees believe that they are in control of the service interaction and can influence the outcome of what they do. Therefore, their need for competence is satisfied. Third, extant literature has consistently demonstrated that empowering leadership could improve the leader-subordinate relationship. For example, leaders’ empowering behaviors can strengthen LMX (Ertürk and Albayrak, 2020) and subordinates’ trust in their leaders. Thus, empowerment by their leaders could satisfy frontline employees’ need for relatedness. Taken together, We propose:

Hypothesis 3: Empowering leadership exerts a positive influence on harmonious passion.

Hypothesis 4: Empowering leadership exerts a negative influence on obsessive passion.

#### 2.2.2 Job passion and emotional labor

Regarding the discretion to regulate one’s emotions, exploring the motivational antecedents of frontline employees’ emotional labor strategies is critical (Busoi et al., 2022). Sisley and Smollan (2012) argued that employees’ autonomous motivation is more likely to predict deep acting, whereas controlled motivation tends to lead to surface acting. Consequently, whether frontline employees utilize deep or surface acting in service encounters depends on which type of their job passion is aroused.

First, employees who exhibit high levels of harmonious passion tend to perform their work with high engagement (Teng et al., 2021). Consistent with this view, in the service context, harmonious passionate employees are motivated to invest more efforts in proactively changing what they feel and displaying authentic emotions (deep acting), rather than just pretending to feel the desired emotions (surface acting). In contrast, frontline employees with obsessive passion would rigidly persist complying organization’s display rules by only faking their emotions, namely surface acting.

Second, previous research has argued that harmonious passion results in individuals experiencing positive emotions. Conversely, obsessive passion would result in individuals experiencing negative emotions (see the meta-review by Pollack et al. (2020)). When interacting with customers, frontline employees are expected to provide excellent service experience by expressing positive emotions (Li et al., 2021). For frontline employees with positive emotions, it is easy for them to meet the organization’s expectation by genuinely expressing their emotions (deep acting). Meanwhile, frontline employees with negative emotions tend to meet the organization’s expectation by hiding their inner feelings and feigning unfelt emotions (surface acting).

Finally, as outlined by Perrewé et al. (2014), job passion, predicts employees’ consistent work intentions and behaviors. Harmonious passionate employees voluntarily integrate job roles into their identity. Under these conditions, intrinsic motivation is evoked (Perrewé et al., 2014), which predicts of employees’ discretionary behaviors (Ho et al., 2018). When interacting with customers, harmonious passionate employees tend to internalize their job requirements and proactively regulate their emotions (deep acting) to ensure desired job outcomes. Conversely, obsessive passionate frontline employees internalize the display rules through a controlled process (Humphrey et al., 2015). For instance, service workers might only display fake positive emotions (surface acting) for fear of customer complaints or punishment. Through a survey of restaurant service workers, Chen et al. (2019) have empirically shown that harmonious passionate employees tended to engaging deep acting. Conversely obsessive passionate employees utilized more surface acting.

Taken together, we propose:

Hypothesis 5: Harmonious passion exerts a positive influence on deep acting.

Hypothesis 6: Harmonious passion exerts a negative influence on surface acting.

Hypothesis 7: Obsessive passion exerts a negative influence on deep acting.

Hypothesis 8: Obsessive passion exerts a positive influence on surface acting.

**FIGURE 1:**
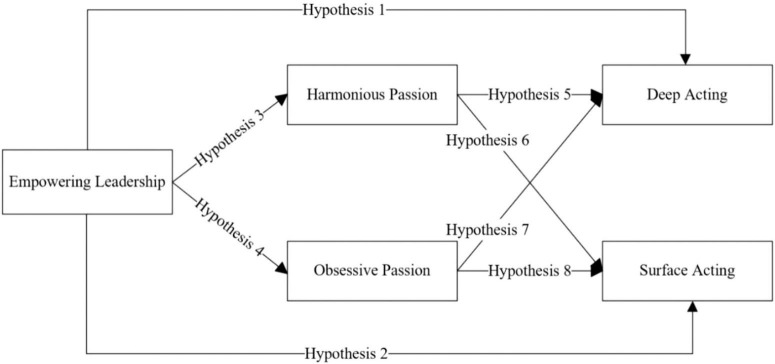
The conceptual model.

Taking all hypotheses together, the Figure 1 shows the conceptual model of this study.

## 3 Materials and methods

### 3.1 Sample and procedures

In consideration of the core variables of the current study, we selected three intensive customer interaction service industries—retail, banking and hospitality—for data collection. In these industries, frontline employees represent service organizations to deliver services in a face-to-face manner. Managing their emotions is therefore very common and necessary in their daily work. In order to ensure an adequate response rate, we turn to MBA alumni who are working in these three industries for help. Finally, two supermarkets, three banks and two hotels were selected for data collection.

At the shift meeting, we invite all service staff whose job involves interacting with customers to participate in this anonymous survey. At the beginning, informed consent (in Chinese) was given to each participant. The purpose of the survey, the procedure and the potential risks were carefully explained. The survey was completely voluntary and anonymous and would take about 15 min to complete. If participants decided to take part in the survey, they were given a questionnaire and an empty envelope. The confidentiality of the participants was ensured by not collecting any sensitive personal information and by returning the questionnaire in a sealed envelope. Once the survey was completed, every participant could receive a gift worth 10–15 RMB. In the end, 1,365 questionnaires were returned. After deleting 325 incomplete responses, the final dataset contained 1,040 usable responses, indicating a response rate of 76.19%. Among the respondents, 55.8% were female and 44.2% were male. For education and experience, 86.9% of respondents had at least a university degree and over 60% respondents tenured for more than 3 years.

### 3.2 Measure

We utilized five-point Likert scales to measure all variables. Empowering leadership was measured using a 12-item scale proposed by Zhang and Bartol (2010) based on Ahearne et al. (2005). Sample item was “The leader trusts me in dealing with customer’s requirements.” The Cronbach alpha of empowering leadership is 0.876. We assessed job passion using the six-item scale proposed by Trépanier et al. (2014). Specifically, there were three items for harmonious passion, and the example item was “My job is in harmony with other aspects of my identity.” Obsessive passion was measured with three items. The example item was “I have a sense of being under the control of my job.” The Cronbach alpha of harmonious passion and obsessive passion are 0.901 and 0.882, respectively. To measure the level of frontline employees’ emotional labor, a six-item scale proposed by Brotheridge and Lee (2003) was utilized. For deep acting (three items), the sample item was “To satisfy the requirements of my job, I spend efforts in adjusting my emotions I must to display.” The Cronbach alpha for deep acting is 0.880. On the other hand, for surface acting (three items), the sample item was “I usually feign emotions that I don’t really experience” and the Cronbach alpha of surface acting is 0.901. As the original measurement scales were developed in English, according to Brislin (1970), we used the translation and back-translation procedure to prepare the questionnaire.

### 3.3 Descriptive statistics, correlations and reliability

Table 1 contains the means, standard deviations, average variance extraction (AVE), specific correlations between these variables and Cronbach alpha coefficients. The Cronbach alpha coefficients of all constructs (ranging from 0.876 to 0.901) are above the threshold of 0.70 (Bland and Altman, 1997), which means that the reliabilities of all measures are acceptable.

**TABLE 1:** Descriptive statistic.

Variables	Mean	SD	AVE	1	2	3	4	5
1. Empowering leadership	3.404	0.787	0.566	**0.876**	–	–	–	–
2. Harmonious passion	3.280	1.072	0.754	0.566***	**0.901**	–	–	–
3. Obsessive passion	3.050	1.122	0.718	0.229***	–0.205***	**0.882**	–	–
4. Deep acting	3.336	1.043	0.716	0.478***	0.561***	–0.179***	**0.880**	–
5. Surface acting	3.067	1.171	0.754	–0.267***	–0.233***	0.209***	–0.121***	**0.901**

The bold diagonal values represent the Cronbach alpha values of core variables. *n* = 1,040; *p < 0.05; **p < 0.01; ***p < 0.001.

### 3.4 Confirmatory factor analysis

Before conducting confirmatory factor analyses, we tested the normality of each variable (Table 2).

**TABLE 2:** Normality test.

Variables	Skewness		Kurtosis	
	**Statistic**	**Std. error**	**Statistic**	**Std. error**
Empowering leadership	–0.424	0.076	–0.099	0.152
Harmonious passion	–0.275	0.076	–0.451	0.152
Obsessive passion	–0.213	0.076	–0.836	0.152
Deep acting	–0.360	0.076	–0.414	0.152
Surface acting	–0.316	0.076	–0.765	0.152

The results showed that the maximum value for the absolute value of skewness of all the variables was 0.424, which was below the threshold value of two. In term of Kurtosis value, the maximum for the absolute value (0.836) was below the threshold value of 7. Therefore, the normality of all variables was acceptable. We conducted confirmatory factor analyses by Amos 28.0 to examine the distinctiveness of core variables (e.g., empowering leadership). The fitness indexes (shown in Table 3) indicated that the data exhibited a reasonable fitness to the 5-factor model: χ^2^/df = 2.683, RMSEA = 0.040, SRMR = 0.031, NFI = 0.974, CFI = 0.983, TLI = 0.979. The comparing of competitive models (Table 3) demonstrated that the five-factor model fitted the data best, suggesting that the validity of all constructs was acceptable.

**TABLE 3:** Results of confirm factor analyses.

Models	χ^2^	d*f*	χ^2^/d*f*	RMSEA	SRMR	NFI	CFI	TLI
5-factor: EL, HP, OP, DA, SA	252.226	94	2.683	0.040	0.031	0.974	0.983	0.979
4-factor: EL, HP + OP, DA, SA	2865.639	98	29.241	0.165	0.200	0.703	0.710	0.645
3-factor: EL, HP + OP, DA + SA	4759.047	101	47.119	0.211	0.241	0.508	0.512	0.420
2-factor: EL + HP + OP + DA, SA	5038.027	103	48.913	0.215	0.175	0.479	0.483	0.398
1-factor: EL + HP + OP + DA + SA	5431.384	104	52.225	0.222	0.181	0.438	0.442	0.356

*n* = 1,040; EL, empowering leadership; HP, harmonious passion; OP, obsessive passion; DA, deep acting; SA, surface acting.

### 3.5 Common method variance

As we used self-report questionnaires to collect the data, the common method variance should be considered. First, we performed the Harman’s one-factor test and found that the largest factor accounted for only 31.46% of the variance (less than cut-off value of 40%), which means that the common method variance is acceptable (Podsakoff et al., 2003). Furthermore, following the suggestion of Williams and McGonagle (2016), a series of comprehensive CMV tests were carried out by comparing several competitive models. According to the procedure, we compared the baseline model, Method U, Method I and Method R (Table 4). The results of Chi-square differences between these four models, according to Williams and McGonagle (2016), indicated that the common method variance in our research would not lead to a bias in the substantive relationships.

**TABLE 4:** Results of common method variance.

Models	χ^2^	d*f*	χ^2^/d*f*	RMSEA	SRMR	CFI	TLI
Baseline	252.226	94	2.683	0.040	0.031	0.983	0.979
Method U	176.114	78	2.258	0.035	0.027	0.990	0.984
Method I	219.417	89	2.465	0.038	0.027	0.986	0.982
Method R	173.668	88	1.974	0.031	0.029	0.991	0.988
**Δ Models**				**Δ χ^2^**	**Δ d*f***	**χ^2^ threshold value at 0.001**	
1. Baseline vs. Method U				76.112***	16	39.25	
2. Method U vs. Method I				43.303***	11	31.26	
3. Method U vs. Method R				2.446	10	29.59	

***p < 0.001.

## 4 Results

According to Hayes (2017), the proposed conceptual model was empirically tested through the PROCESS macro (Table 5). First, we tested how empowering leadership influences deep acting via job passion. Hypothesis 1 claimed that empowering leadership would facilitate deep acting. The results of Model 1 confirmed a positive relationship between empowering leadership and deep acting (β = 0.358, *p* < 0.001), supporting Hypothesis 1. Furthermore, the results of Model 2 indicated that empowering leadership positively and significantly affected harmonious passion (β = 0.570, *p* < 0.001). Hypothesis 3 was therefore supported. However, contrary to Hypothesis 4, the coefficients of Model 3 indicated that empowering leadership predicted obsessive passion (β = 0.228, *p* < 0.001). Hypothesis 4 was therefore rejected. In Model 4, deep acting was simultaneously regressed on empowering leadership, harmonious passion and obsessive passion. The coefficients of Model 4 indicated that the positive influence of empowering leadership on deep acting remained (β = 0.110, *p* < 0.01). Harmonious and obsessive passion had a positive (β = 0.477, *p* < 0.001) and negative (β = –0.103, *p* < 0.001) relationship with deep acting, respectively. Hypotheses 5 and 7 were supported.

**TABLE 5:** Test of mediating effects.

Independent variable		Model 2: harmonious passion		Model 3: obsessive passion		Model 1: deep acting		Model 4: deep acting		Model 5: surface acting		Model6: surface acting	
		**β**	**SE**	**β**	**SE**	**β**	**SE**	**β**	**SE**	**β**	**SE**	**β**	**SE**
Control variables	Gender	0.025	0.056	0.045	0.069	–0.007	0.062	–0.014	0.054	0.031	0.073	0.023	0.071
	Age	0.038	0.033	–0.014	0.041	0.086	0.036	0.066	0.032	–0.011	0.043	–0.004	0.042
	Education	–0.026	0.033	0.023	0.041	–0.023	0.037	–0.009	0.032	–0.039	0.044	–0.046	0.042
	Tenure	–0.032	0.027	0.028	0.034	–0.062	0.030	–0.044	0.026	0.006	0.036	–0.004	0.034
	Industry 1	0.019	0.047	–0.003	0.094	0.037	0.083	0.028	0.073	–0.022	0.099	–0.020	0.096
	Industry 2	0.002	0.005	–0.010	0.080	0.044	0.071	0.042	0.063	–0.019	0.085	–0.017	0.082
Empowering leadership	0.570***	0.032	0.228***	0.039	0.358***	0.035	0.110**	0.042	–0.160***	0.042	–0.154***	0.043	
Harmonious passion	–	–	–	–	–	–	0.477***	0.033	–	–	–0.101	0.054	
Obsessive passion	–	–	–	–	–	–	–0.103***	0.027	–	–	0.224***	0.035	
R^2^	0.324	0.056	0.134	0.330	0.029	0.099							

*n* = 1,040; *P < 0.05 **p < 0.01; ***p < 0.001.

To test the mediating role of job passion in the relationship between empowering leadership and deep acting, we conducted a bootstrap analysis. The coefficients (Table 6) of the bootstrapping sample provided support for the mediating role of harmonious passion (0.271, 0.393) in the empowering leadership-deep acting link. In addition, regarding the mediating role of obsessive passion in the relationship between empowering leadership and deep acting, the coefficients (Table 6) of the bootstrapping sample also confirmed that empowering leadership indirectly affected deep acting via obsessive passion (–0.051, –0.010).

**TABLE 6:** Coefficients of bootstrap analyses.

Path		Effect	Boot SE	95% confidence interval	
				**Lower**	**Upper**
Empowering leadership → deep acting	Total effects	0.433	0.035	0.364	0.502
	Direct effects	0.132	0.042	0.051	0.214
	Indirect effects via harmonious passion	0.329	0.031	0.271	0.393
	Indirect effects via obsessive passion	–0.029	0.011	–0.051	–0.010
Empowering leadership → surface acting	Total effects	–0.217	0.042	–0.299	–0.135
	Direct effects	–0.208	0.054	–0.314	–0.102
	Indirect effects via harmonious passion	–0.078	0.041	–0.158	0.002
	Indirect effects via obsessive passion	0.070	0.016	0.041	0.102

Secondly, we examined how empowering leadership affected surface acting via job passion. Specifically, the results of Model 5 predicted that empowering leadership would result in a decrease in surface acting (β = –0.160, *p* < 0.001). Therefore, hypothesis 2 was supported. Furthermore, the coefficients of Model 6 indicated that the relationship between empowering leadership and surface acting remained significant (β = –0.154, *p* < 0.001). In addition, obsessive passion would increase surface acting (β = 0.224, *p* < 0.001). However, the coefficient didn’t support the effect of harmonious passion on surface acting. Thus, the results confirmed the support of Hypothesis 8 and the rejection of Hypothesis 6.

The results (reported in Table 6) of the bootstrap analyses confirmed the mediating role of harmonious and obsessive passion in the link between empowering leadership and surface acting. Specifically, the indirect link from empowering leadership to surface acting via obsessive passion (0.041, 0.102) was positive and significant. In contrast, the indirect influence of empowering leadership on surface acting through harmonious passion (-0.158, 0.002) was rejected.

**FIGURE 2:**
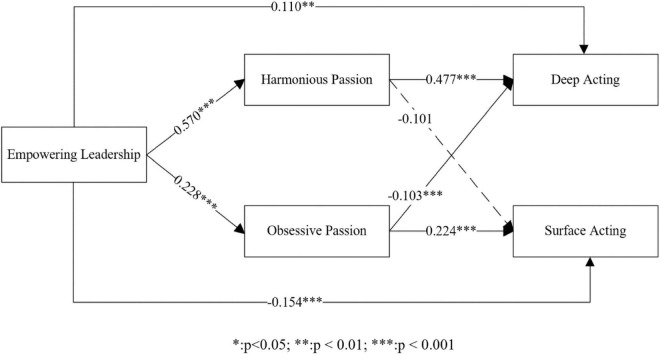
Result of hypothesized mediation model.

Taken together, all hypotheses were supported with the exception of hypotheses 2 and 6 (Figure 2). Empowering leadership could predict an increase in deep acting and a decrease in surface acting. In addition, harmonious passion and obsessive passion act as parallel mediators of these effects.

## 5 Discussion

From the perspective of job passion, we explored the process of how empowering leadership was internalized and, in turn, elicited different emotional labor strategies. The empirical findings indicated that empowering leadership facilitated frontline employees’ deep acting, while hindered their surface acting. Furthermore, there were two inconsistent indirect paths from empowering leadership to deep acting. The positive indirect path was via harmonious passion, while the negative indirect path was via obsessive passion. Regarding the indirect influence of empowering leadership on surface acting, only obsessive passion served as a partial mediator. Contrary to our prediction, empowering leadership led to an increase, but not a decrease, in obsessive passion of frontline employees.

### 5.1 Theoretical implications

The present research provides empirically grounded insights into how empowering leadership affects emotional labor through job passion. The findings contribute to previous research in three ways. First, our findings advance the knowledge of the outcomes of leader’s empowering behavior by testing its influence on frontline employees’ emotional labor. Although Kim et al. (2018) called for investigating how empowering leadership affects subordinates’ emotions in the future, as far as we know, little previous research has provided evidence on how employees generate emotional responses to empowering leadership. Based on the research results, we conclude that empowering leadership can influence the way frontline employees regulate their emotions. Moreover, in recent years, the spillover effects of empowering leadership on customers have attracted the interest of scholars (Lin et al., 2019). Especially in the service industry, the boundary-spanning role of frontline employees makes their emotion regulation important in shaping customer experience. Our findings provide evidence that the spillover effects of leaders’ empowering behaviors can reach customers through frontline employees’ emotional labor. Furthermore, the assumption of reciprocity is not valid when employees’ job behaviors target customers rather than their leaders. Therefore, different from previous studies relying on SET or LMX to explain why employees’ response empowering leadership by their job behaviors (Horoub and Zargar, 2022; Rai and Kim, 2021), our findings explored the connection between empowering leadership and frontline emotional labor from a motivational perspective, which provide a new theoretical foundation.

Second, drawing upon the job passion model, this study proposed parallel mediators to integrate the inconsistent conclusion of the influence of empowering leadership on subordinates. From the job passion perspective, we proposed a psychological mechanism to explain how employees internalize leadership style and in turn foster their job behaviors. Most previous studies acknowledged the beneficial outcomes of empowering leadership, including facilitating subordinates’ proactive behaviors and improving positive job attitudes. However, the “dark side” of leaders’ empowering behavior has been recognized in recent studies. Undesirable outcomes of empowering leadership include emotional exhaustion (Li et al., 2023), unethical behavior (Dennerlein and Kirkman, 2022) and job tension (Cheong et al., 2016). Particularly in China, with high power distance and collectivism, researchers have argued that the effectiveness of empowerment was lower than in Western countries with low power distance and individualism (Eylon and Au, 1999). Our findings support this view by confirming the “dark side” effects of empowering leadership exist in service encounters. Service workers are socialized to work under conditions of limited information, low responsibility, and much more guidance with high power (Li et al., 2017). Once they are empowered with job autonomy and responsibility, they may experience job stress, such as role ambiguity (Humborstad and Kuvaas, 2013). Under these conditions, empowered frontline employees may persist in their jobs with obsessive passion. Furthermore, the present research showed that leaders’ empowering behaviors exert “double-edged” effects on their subordinates’ emotional labor through different mechanisms. For its “bright” effects, empowering leadership promotes harmonious passion, which in turn leads to deep acting. Whereas, for its “dark” effects, empowering leadership also facilitates obsessive passion, which further predicts surface acting and decreases deep acting. Thus, leader’s empowering behaviors influence their subordinates’ emotional labor through two distinct psychological mechanisms. Consistent with previous literature that views empowerment as a “double-edged sword” (Li et al., 2023), our findings make contributions by providing a framework to integrate mixed evidence of the consequence of leader’s empowering behaviors.

Third, our findings extend the understanding of how frontline employees internalize their perceived leadership style and further engage in different emotional labor strategies. Although both practitioners and researchers have realized the importance of frontline employees’ emotional labor, it is challenging for leaders to intervene and monitor frontline employees who conduct deep or surface acting in service interactions. The results of this study not only enrich the antecedents of emotional labor but also supply management tools for service managers to guide frontline employees’ emotional labor. Furthermore, although Cheong et al. (2019) argued that empowerment could enhance employees’ internal motivation, which was considered as predictor of frontline employees’ emotion regulating behaviors (Gabriel et al., 2016), it is not clear which specific motivational constructs could serve as mediators. By confirming job passion’s mediating effects during the link from empowering leadership to emotional labor, the present study sheds light on how leaders can effectively intervene or guide their subordinates to choose distinct emotional labor strategies. Finally, grounded in the Conservation of Resources (COR) theory, we establish the theoretical foundation for the direct effect of empowering leadership on emotional labor. From the perspective of work resources, we further investigate the mechanisms through which empowering leadership influences employee emotional labor, thereby contributing to the literature on the impact mechanisms of empowering leadership on employee behaviors.

### 5.2 Practical implications

Given the dynamic nature of service, sufficient autonomy is required to meet the diverse needs of customers. The current research found that empowering leadership was an important way for service managers to influence frontline employees’ emotional labor. Therefore, service organizations could draw several practical implications from these findings.

Service managers responsible for frontline employees should be encouraged to carry out empowering leadership. First, when hiring or promoting service managers, empowering leadership should be considered as a critical selection factor. Preference should be given to candidates who admire empowerment and have demonstrated empowering leadership in their previous work experience. Second, service organizations could train their managers in specific empowerment skills. Such as inviting subordinates to participate in decision making (Tang et al., 2020), sharing information (Syahrul, 2020), and expressing confidence in high performance (Audenaert and Decramer, 2018). Specifically, given the interactive nature of service, service managers could grant frontline employees enough job autonomy by encouraging them to craft their interactions with customers and provide personalized service. In addition, service managers could involve frontline employees in decision making by encouraging and accepting their suggestions for improving the service process.

The limits of empowerment also need to be recognized by service managers. According to our findings, the “dark side” of leader’s empowering behavior on subordinates’ emotional labor is illustrated by the impairment of deep acting through obsessive passion. As Lee et al. (2017) argued, empowering leadership involves high responsibility which may lead to subordinates’ job pressure. In particular, if frontline employees do not have the competence to effectively utilize the empowered authority, they would perceive empowerment as a kind of job burden. Therefore, service organizations need to consider the effectiveness of empowering leadership from the standpoint of frontline employees through surveys or interviews. If frontline employees perceive empowerment as a burden that “forces” them to work, service managers should provide additional job resources or guidance to help frontline employees achieve high performance. Further, in China with high power distance, service workers used to follow the rules set by the hierarchy. Before empowering, service managers should differentiate subordinates according to their willingness or attitude.

As the competition of service industry goes fiercer, service organizations should pay attention to frontline employees’ job passion. Our empirical results indicate that job passion has an essential mediating function in how frontline employees internalize their leader’s empowering behaviors and, in turn, respond by using different strategies to regulate their emotions in service encounters. Specifically, harmonious passionate frontline employees tend to manage their internal feelings and express authentic emotions (deep acting). Whereas, obsessive passionate frontline employees would express fake emotions (surface acting) to meet the organization’s requirement. Therefore, to ensure the effectiveness of empowering leadership, it is necessary for service organizations to take measures to evoke harmonious passion in frontline employees and reduce their obsessive passion. In addition to empowerment from their leaders, flexible workplace gives frontline employees opportunity to perform job crafting. Prior research has revealed that job crafting could foster harmonious passion (Teng, 2019; Yadav and Dhar, 2021).Therefore, service organizations could create a flexible environment by providing adequate job resources (Trépanier et al., 2014) and organizational supports (Nasution and Amalia, 2024). In addition, service organizations could evoke frontline employees’ harmonious passion by satisfying their basic psychological needs. Besides frontline employees’ need for autonomy, which can be satisfied through empowering leadership, their needs for competence and relatedness should also be considered. Previous research has shown that providing constructive feedback was a helpful way to cultivate harmonious passion (Ho et al., 2011). Therefore, service managers should provide timely positive feedback to confirm the competence of frontline employees. In the Chinese culture, admiration from senior management sends a strong signal to frontline employees about their work competence. An important way to satisfy frontline employees’ psychological need for relatedness is to develop high quality leader-member exchange (LMX). Frontline employees with high LMX tend to experience high relatedness to the organization, which in turn enhances their harmonious passion.

### 5.3 Limitations and future research direction

Notwithstanding these significant contributions, several limitations warrant careful consideration and should be addressed in future research endeavors. First, we collected the data by inviting frontline employees to complete the questionnaires. This cross-sectional data cannot capture the variance of frontline employees’ emotional labor between different working days (Wang et al., 2021). Therefore, in follow-up research, experience sampling method can be used to collect data and multilevel analyses can be conducted to test the wintin-individual variance of emotional labor. Furthermore, although the CMV of this cross-sectional data is acceptable, we also suggest that the survey should be designed to collect data in a time-lagged manner in future research.

Second, the research only collect data in China. Given the different cultures of Western and Asian countries, which limit the universality of our findings. Kim et al. (2018) have argued that the effectiveness of leaders’ empowering behavior would be weak in Asian countries with high power distance and collectivism. The future study could consider the cultural differences between Asian and Western countries by introducing cross-cultural variables, including power distance and individualism–collectivism. Our recommendation is to collect data from several countries, the universality of the research findings will be increased.

Finally, the boundary condition of our findings should be explored in the future. The findings of this study demonstrated the coexistence of the “bright side” and “dark side” effects of empowering leadership. However, the question remains: under what specific conditions do the “bright side” and “dark side” of empowering leadership become more predominant? The answer is not clear. We suggest that the boundary conditions of these relationships should be investigated in the future. For example, according to the person–environment fit, the effectiveness of empowering leadership depends on workplace environment and individual differences (Kim and Beehr, 2018; Vergauwe et al., 2022). Therefore, the personality traits and features of the workplace environment, such as the organizational climate, are expected to influence the type of job passion is elicited by empowering leadership.

## Data Availability

The original contributions presented in the study are included in the article/supplementary material, further inquiries can be directed to the corresponding author.
